# Efficacy and safety of acupuncture for patients with pseudomyopia: A protocol for systematic review and meta-analysis

**DOI:** 10.1097/MD.0000000000028859

**Published:** 2022-02-11

**Authors:** Ye Niu, Wei Zheng, Shan Wang, Qi Zhao, Lijuan Wei, Yang Zhao, Bo Wang, Yuan Ju, Fuchun Wang

**Affiliations:** a Changchun University of Chinese Medicine, 1035 Boshuo Road, Changchun City, Jilin Province, China; b Affiliated Hospital of Changchun University of Chinese Medicine, 1478 Gongnong Road, Changchun City, Jilin Province, China; c Department of Acupuncture, The Affiliated Hospital of Changchun University of Chinese Medicine, 1478 Gongnong Road, Changchun City, Jilin Province, China.

**Keywords:** pesudomyopia, protocol, systematic review, thumbtack needle

## Abstract

**Background:**

Myopia are common health problems that people experience in daily life. Pediatric myopia has become a major international public health concern that has a negative impact on physical, mental health of patients, and quality of life. Currently, there is no cure available. Press needle as an adjuvant therapy is currently undergoing clinical trials in different medical centers. However, no relevant systematic review or meta-analysis has been designed to evaluate the effects of press needle patching on early myopia. There is also a lack of systematic evaluation and analysis of acupoints and thumbtack needle.

**Methods:**

We will electronically search Medline, Embase, PubMed, Web of Science, the Cochrane Central Register of Controlled Trials, China National Knowledge Infrastructure, Chinese Biomedical Literature Database, Chinese Scientific Journal Database, and Wan-Fang Database from their inception to October 2021. In addition, we will manually retrieve other resources including the reference lists of identified publications, conference articles, and gray literature. The clinical randomized controlled trials or quasi-randomized controlled trials related to press needle for the treatment of early myopia will be included in the study. The language is limited to Chinese and English. Research selection, data extraction, and research quality assessment will be independently completed by 2 researchers. Data were synthesized by using afixed effect model or random effect model depend on the heterogeneity test. The total effective rate was the primary outcomes. RevMan V.5.3 statistical software will be used for meta-analysis. If it is not appropriate for a meta-analysis, then a descriptive analysis will be conducted. Data synthesis will use the risk ratio and the standardized or weighted average difference of continuous data to represent the results.

**Results:**

This study will analyze the clinical effective rate, pesudomyopia outcomes, quality of life, improvement of clinical symptoms of pesudomyopia, and validity of thumbtack needle for patients with pesudomyopia.

**Conclusion:**

This systematic review will provide evidence to judge whether thumbtack needle is an effective intervention for patients with early myopia.

**Systematic review registration:**

PROSPERO, CRD42021243151.

## 1 Introduction

Pseudomyopia is caused by the continuous contraction and spasm of the ciliary muscle due to excessive use of the eye, the thickness of the lens increases, and the vision is blurred. Accommodation involves changing the optical power to sharply focus on objects placed at varying distances. Although accommodation can be consciously controlled, it usually acts as a reflex. In response to accommodation stimuli, the ciliary muscles contract to thicken the lens, and convergence and miosis occur.^[1]^ Excessive accommodation, pseudomyopia, can occur separately, and result in blurred distance vision due to increased refractive power generated by ciliary muscle spasm, asthenopia, and headache.^[2–6]^ In order to adapt to this situation, the length of the eye axis will lead to the formation of true myopia. In recent years, the incidence of myopia has increased day by day, and myopia has become a child. One of the main causes of visual impairment. At present, myopia—a public health problem.^[7]^ Researchers found that the global prevalence of myopia is about 28.3%, and it is increasing sharply. It is estimated that by 2050, half of the world's population will suffer from myopia.^[8]^ Therefore, effective treatment of pseudomyopia is the fundamental reason to prevent the development of myopia.

At present, the treatment of the disease mainly includes conventional treatment, drug treatment, biofeedback treatment, and surgical treatment, but the therapeutic effects vary and are often unsatisfactory. Pharmacological agents such as 0.01% atropine are being prescribed in Asia, and show a reduction of up to 50% in the rate of progression of myopia, although there is no reduction in the rate of axial elongation. Oral tablets containing 7-methylxanthine (7-MX) have been approved for use in children in Denmark for myopia control and shows some efficacy, but long-term studies are needed.^[9]^ While these approaches have been tried in isolation, they may have a greater impact if different approaches are combined. Uncorrected refractive errors are the leading cause of moderate to severe visual impairment globally and the second cause of blindness worldwide.^[10]^ Therefore, many people, including those who do not improve with existing medications or suffer many side effects, are interested in complementary and alternative medicine.

Thumbtack needle belongs to the category of acupuncture and is an ancient Chinese medicine method in which acupuncture points on the skin are manually stimulated with thumbtack needle. Thumbtack needle is a treatment method to prevent diseases by strengthening the immune system through the stimulation of acupuncture points with microacupuncture.^[11]^ Thumbtack needle are applied to acupoints, such as BL-2, HN-5, and HN-4, for 4 to 6 hours.^[12]^ The earliest record of thumbtack needle can be traced back to the classic “Ling shu· Nine Needles Theory” (Lingshu Jiuzhenwen) where thumbtack needle was listed as a treatment method, and it is still widely used today.^[13]^ Modern studies suggest that thumbtack needle can regulate the level of related asthenopia peptides while improving the ciliary muscle, thereby functioning in the treatment of the visual system diseases.^[14]^ As a complementary and alternative therapy, thumbtack needle is often used to treat chronic functional diseases, and its application in myopia has gradually become popular in recent years.^[15,16]^ However, the clinical efficacy and potential treatment prescriptions of thumbtack needle for pesudomyopia remain unclear, requiring further exploration. Therefore, a systematic evaluation of treatment outcomes and treatment prescriptions may help better explain and push this method into practice. In this study, we will investigate current evidence associated with the effectiveness and safety of thumbtack needle for pesudomyopia, which will help clinicians to better use it in clinical practice.

## 2 Methods

### 2.1 Study type

We will collect randomized controlled trials (RCTs) to evaluate clinical effectiveness, functional outcomes, quality of life, and side effects of thumbtack needle on pesudomyopia for systematic review and meta-analysis. RCTs comparing thumbtack needle for pesudomyopia with no treatment, placebo, or conventional drugs (e.g., mydriatic drugs) will be included. All eligible trials will be included regardless of language and publication type. RCTs that meet the requirements will be included for data mining. Articles of the following research types will be excluded: case series, observational studies (including cohort studies and case-control studies) and retrospective studies, qualitative studies, animal experiments, review articles. In addition, there will be no restrictions on study area, race, patient age, and gender.

### 2.2 Participants

This review will include patients of any age who had been diagnosed with pesudomyopia without limitations related to gender, race, study area, and education status. The diagnosis of pseudomyopia requires a visual inspection and a cycloplegia inspection. Exclude if they are diagnosed with true myopia and pathological myopia.

### 2.3 Interventions

Participants in the intervention group are those thumbtack needle, regardless of thumbtack needle length, acupoints selected, stimulation time. There will not be any restrictions on age and original countries of the participants. In the control group, patients received medication, no treatment, sham or placebo acupoint catgut embedding, acupuncture/electro-acupuncture, and etc. The other interventions between the control group and the intervention group should be the same.

### 2.4 Outcome measures

The primary outcomes will include visual outcome, visual inspection results after mydriasis, axial length inspection result, corneal thickness measurement results, and clinical effective rate. The secondary outcomes will be adverse events and discontinuations due to adverse events.

### 2.5 Search strategy

An electronic search will be conducted. We will identify relevant studies from the Cochrane Central Register of Controlled Trials, PubMed, Embase, the Web of Science, the Chinese Biomedical Literature Database (CBM), the Chinese Scientific Journal Database (CSJD), the Wan-Fang Database (Wanfang), and the China National Knowledge Infrastructure (CNKI) from their inception to November 15, 2021. The search term will consist of 3 parts: intervention method, disease, and study type: (“acupoint application” or “acupuncture” or “embedding acupuncture” or “ thumbtack needle” or “embedded needles” or “external application therapy” or “needle embedding therapy” or “thumb tack needle for subcutaneous embedding” or “embedding technique” or “acupuncture point application therapies” or “acupoint stimulation therapy”) and (“pseudomyopia” or “early myopia”) and (“randomized controlled trial” or “randomized” or “case control studies” or “observational studies” or “case series” or “trial”), and (“blind”). The details of the PubMed and Wan-Fang Database search strategies are provided in Tables 1 and 2. The similar but adaptive search strategies will be applied to other electronic databases. Language will be restricted to English and Chinese. Reference lists of relevant original studies will be screened to identify additional potentially citations. In addition, the following 3 trial registries will be searched for ongoing studies: Current Controlled Trials: www.controlled-trials.com; Clinical Trials: www.ClinicalTrials.gov; and Chinese Clinical Trial Registry: www.chictr.org.cn/index.aspx.

**Table 1:** The search strategy for PubMed database.

Number	Search terms
#1	press needle [MeSH]
#2	thumbtack needle [MeSH]
#3	cutaneous needle [MeSH]
#4	skin needle [MeSH]
#5	dermal needle [MeSH]
#6	microneedle [MeSH]
#7	raphide [MeSH]
#8	trichodragma [MeSH]
#9	fine needle thermistor [MeSH]
#10	microneedle therapy system [MeSH]
#11	needle embedding therapy [MeSH]
#12	down picker [MeSH]
#13	intradermal imbedding needle [MeSH]
#14	intradeamal needle [MeSH]
#15	needle embedding [MeSH]
#16	complementary and alternative medicine [MeSH]
#17	auricular acupuncture [MeSH]
#18	#1 or #2 or #3 or #4 or #5 or #6 or #7 or #8 or #9 or #10 or #11 or #12 or #13 or #14 or #15 or #16 or #17
#19	nearsightedness [MeSH]
#20	pseudomyopia [MeSH]
#21	#19 or #20
#22	randomized controlled trial [MeSH]
#23	case control studies [MeSH]
#24	observational studies [MeSH]
#25	case series [MeSH]
#26	trial [MeSH]
#27	#22 or #23 or #24 or #25 or #26
#28	#18 and #21 and #27

**Table 2:** The search strategy for Wanfang database.

Number	Search terms
#1	press needle [MeSH]
#2	thumbtack needle [MeSH]
#3	cutaneous needle [MeSH]
#4	skin needle [MeSH]
#5	dermal needle [MeSH]
#6	microneedle [MeSH]
#7	raphide [MeSH]
#8	trichodragma [MeSH]
#9	fine needle thermistor [MeSH]
#10	microneedle therapy system [MeSH]
#11	needle embedding therapy [MeSH]
#12	down picker [MeSH]
#13	intradermal imbedding needle [MeSH]
#14	intradeamal needle [MeSH]
#15	needle embedding [MeSH]
#16	auricular acupuncture [MeSH]
#17	#1 or #2 or #3 or #4 or #5 or #6 or #7 or #8 or #9 or #10 or #11 or #12 or #13 or #14 or #15 or #16
#18	nearsightedness [MeSH]
#19	pseudomyopia [MeSH]
#20	#18 or #19
#21	randomized controlled trial [MeSH]
#22	case control studies [MeSH]
#23	observational studies [MeSH]
#24	case series [MeSH]
#25	trial [MeSH]
#26	#21 or #22 or #23 or #24 or #25
#27	#17 and #20 and #26

### 2.6 Study selection and data extraction

**Figure 1:**
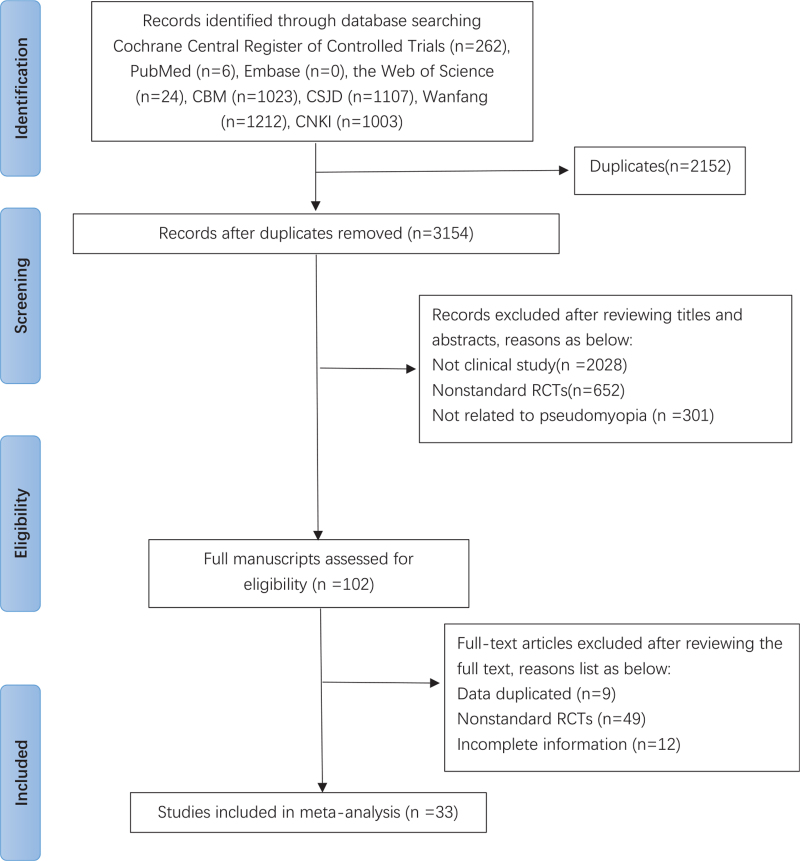
Flow chart of the search process.

Author (WS) with experience in the field will guide the search. First, the NoteExpress 3.2.0 software (Available at: http://www.inoteexpress.com/aegean/) will be used to exclude duplicate references from different databases. Two review authors (NY, ZQ) will independently assess the title and abstracts of all citations found from the above search strategy. A copy of the full text article is obtained for the potentially eligible studies. These review authors will independently read the full text articles to include eligible studies; disagreement will be resolved by consensus through discussion with a third review author (WS). If conclusion still cannot be met, we will contact the author of the article to determine the eligibility of the study. The selection process will be showed in a PRISMA flow chart (http://www.prismastatementorg/) (Fig. 1). In the end, 2 review authors (NY, ZQ) will extract data using a data extraction form according to the recommendations of the Cochrane Handbook for Systematic Reviews of Interventions. The following data will be extracted: author, year of publication, country where the study was conducted, study period, original inclusion criteria, total number of people included in the study, acupoints, stimulus intensity and time of application, and etc.

### 2.7 Addressing missing data or unclear measurement scales

We will try to contact the first or corresponding authors of the included studies to get missing or insufficient trial data by email or telephone. If additional data are not available, we will analyze the existing data, and discuss the potential impact of missing data.

### 2.8 Risk of bias in included studies

Two review authors (NY, ZW) will independently evaluate each included study and will follow the domain-based evaluation as developed by the Cochrane Handbook for Systematic Reviews of Interventions. They will assess the following domains:

1. selection bias (random sequence generation and allocation concealment),
2. performance bias (blinding of participants and personnel),
3. detection bias (blinding of outcome assessment),
4. attrition bias (incomplete outcome data),
5. reporting bias (selective reporting),
6. other bias (such as pre-sample size estimation, early stop of trial).

Each domain will be divided into 3 categories: “low risk,” “high risk,” or “unclear risk.”

### 2.9 Data synthesis and analysis

RevMan V.5.3 (Available at: https://community.cochrane.org/help/tools-and-software/revman-5, provided by The Cochrane Collaboration) statistical software will be applied for data synthesis.^[17]^ A meta-analysis using random or fixed effects models will be conducted to summarize the data. Continuous data will be pooled and presented as mean differences or standardized mean difference with their 95% confidence interval (CI). Dichotomous data will be pooled and expressed as risk ratio with their 95% CI. We will interpret it using the following criteria: *I*^2^ values of 25% is considered low levels of heterogeneity, 50% indicated moderate levels, and 75% indicated high levels.^[18]^ Since low or moderate heterogeneity suggests little variability among these studies, the data will be analyzed in afixed-effects model.^[19]^ When significant heterogeneity occurs among the studies (*P* < .05, *I*^2^ > 50%), a random-effect model will be performed to analyze the data.

### 2.10 Additional analyses

A subgroup analysis will be conducted to explore the potential causes of heterogeneity if necessary. Subgroup analysis will be conducted to evaluate the specific influence of intervention type, age, course of disease, treatment duration on pooled results. If the data are insufficient, qualitative synthesis will be conducted instead of quantitative synthesis. In addition, sensitivity analysis will be performed to examine the robustness of the results by eliminating low quality trials. We will also use Spass software (Version19.0) (Available at: https://www.ibm.com/analytics/spss-statistics-software) for complex network analysis to explore the potential core prescription of thumbtack needle for pseudomyopia.

### 2.11 Assessment of reporting biases

Reporting bias will be evaluated by visual inspection of Funnel plots. At the same time, Begg test and Egger test will be used to test whether the funnel plot is symmetrical. A *P* value <.05 in Egger test or Begg test is considered statistically significant.^[20]^

### 2.12 Confidence in cumulative evidence

In order to better prepare results for usage in guideline development. We will use the Grading of Recommendations Assessment approach to assess the overall quality of evidence supporting the primary outcomes.^[21]^

Five downgrading actors including risk of bias, inconsistency, indirectness, imprecision, and publication bias will be assessed. The assessment results will be divided into 4 levels: high, moderate, low, or very low.

## 3 Discussion

Pseudomyopia is caused by the continuous contraction and spasm of the ciliary muscle due to excessive use of the eye, the thickness of the lens increases, and the vision is blurred.^[2]^ The prevalence of pseudomyopia is reported as approximately 24% in 6-year-olds and 18% in 13-year-olds and is likely to increase further given the amount of time spent by children and adults on electronic devices such as smartphones, tablets, and laptops.^[22,23]^ Pseudomyopia will gradually develop into true myopia or even high myopia, high myopia is associated with an increased risk of developing sight-threatening conditions such as myopic macular degeneration (defined as atrophic changes or choroidal neovascularization in the macular region in high myopia), retinoschisis, posterior staphyloma, glaucoma retinal detachment, and cataract.^[10]^

At present, the treatment of pseudomyopia varies from medical methods. Although lifestyle interventions help some patients with pseudomyopia, there is a lack of data to support their efficacy in those with pseudomyopia. If empirical treatment for patients with pseudomyopia fails,^[24–28]^ cycloplegic drugs (e.g., atropine and homatropine, homide, and tropicamide).^[29]^ Cycloplegic drugs, though effective in improve vision, they are associated with systemic side effects and ocular discomfort. This required tapering of doses until these symptoms were manageable or required switching to weaker cycloplegics.^[30,31]^

Therefore, a number of patients with pseudomyopia attempt to use complementary and alternative therapy, including thumbtack needle. Thumbtack needle is a microneedles that is applied to specific acupoints to stimulate the skin, meridians, and collaterals to produce preventive and therapeutic effects. Pseudomyopia is the most common condition treated by thumbtack needle.

With the development of complementary and alternative medicine, thumbtack needle as a product of the combination of traditional acupuncture and modern medicine, has the characteristics of simple operation, long curative effect, low cost, no obvious side effects. And it is widely used in the treatment of pseudomyopia. Nevertheless, currently, there is not a SR-related to thumbtack needle for pseudomyopia has been published in English. This study will collect evidence comprehensively, extract and analyze the data, and then draw reasonable conclusions, hoping to provide convincing evidence for patients and clinicians during the decision-making process.

## 4 Ethics and dissemination

Ethical approval is not required. Since this work is carried out on published data. We aimed to explore the clinical effective rate, quality of life, improvement of clinical symptoms of pseudomyopia, as well as effective prescription of thumbtack needle for patients with pseudomyopia. In the end, the results will be submitted to a peer-reviewed journal.

## Acknowledgments

The authors appreciate the financial support received from the Natural Science Foundation of Jilin Province (grant number: 202512JC010477466) and scientific research and development fund of Changchun University of Chinese Medicine.

## Author contributions

Ye Niu and Shan Wang had the original idea of this work and drafted the protocol. Qi Zhao and Yang Zhao designed the search strategies. Lijuan Wei proposed some advice for the design and revision. Wei Zheng designed the flow chart. All authors critically revised the draft and approved the final manuscript.

**Conceptualization:** Ye Niu, Wei Zheng.

**Data curation:** Shan Wang, Qi Zhao, Wei Zheng.

**Formal analysis:** Ye Niu, Wei Zheng.

**Funding acquisition:** Lijuan Wei.

**Investigation:** Yang Zhao, Wei Zheng.

**Methodology:** Bo Wang, Yuan Ju.

**Project administration:** Wei Zheng, Lijuan Wei.

**Resources:** Ye Niu, Wei Zheng, Shan Wang, Qi Zhao.

**Software:** Wei Zheng, Fuchun Wang.

**Supervision:** Lijuan Wei.

**Validation:** Ye Niu, Wei Zheng.

**Visualization:** Lijuan Wei.

**Writing – original draft:** Ye Niu, Wei Zheng.

**Writing – review & editing:** Wei Zheng, Yuan Ju, Lijuan Wei.
